# Multiomics and Health: A Holistic Approach to Better Understand the Role of the Microbiome

**DOI:** 10.3390/ijms221910786

**Published:** 2021-10-05

**Authors:** Gary A. Toranzos, Tasha M. Santiago-Rodriguez

**Affiliations:** 1 Environmental Microbiology Laboratory, Biology Department, University of Puerto Rico, San Juan, PR 00931, USA; 2 Diversigen Inc., 3 Greenway Plaza, Suite 1575, Houston, TX 77046, USA

The present Special Issue focuses on the latest approaches to health and public health microbiology using multiomics. Public health microbiology was, for decades, dependent on the isolation and characterization of infectious agents, usually a very long and troublesome path, often leading to Type 1 (false positive) or Type 2 (false negative) errors. Public health microbiology has evolved to demonstrate that most microorganisms, both commensal and pathogenic, cannot be grown in the laboratory (one example is *Treponema pallidum*, the etiological agent of syphilis). We also now know that many infections are usually not the result of a single opportunistic pathogen but are rather polymicrobial infections. We also know that a consortium of microbe–host and microbe–microbe interactions are key in maintaining a healthy state. The new omics approaches to health are giving us a fine-tuned view of the microorganisms present in a sample (environmental or clinical). This omics cascade enables the flow of information from nucleic acids (genome/metagenomes) to gene expression (transcriptome/meta-transcriptomes), and function (proteome/meta-proteomes, and metabolomes) in single- or meta-organisms (i.e., host and microbiome). However, (and this is a big however), we are still in the infancy of this approach as we are lagging on the development, implementation, and use of appropriate analytical tools for data normalization, analysis, and comparisons within and across omics datasets. Despite this, a multiomics approach is opening a possibility for public health surveillance, diagnosis, as well as personalized treatment, with almost lightning speed, ... ok, slow lightning, but compared to the past, these approaches can be carried out with plenty of time to diagnose and treat a patient, or take remedial actions in the case of the environment. 

This is the case of microbiome transplantations. Fecal transplants, for instance, although touted as a recently discovered method to treat gastrointestinal problems, is in fact an old remedy, in cows, anyway, where the cud, or rumen contents of a healthy cow were given to sick cows with apparently good results. A very similar procedure is being done with humans, with great success. Omics studies on the fecal inoculant ensure a safe procedure, as well as a standardized inoculum. In addition, the application of omic techniques enables following the patient’s gut microbiome over time to ensure a successful transplantation and adaptation. Similarly, the latest studies on the microbiome of the skin give us a glimpse on possible future skin microbiome transplants, including, may we presume, dental transplants that may make caries a thing of the past, as well as transplantation of a healthy skin microbiome and/or prebiotics and postbiotics. Notably, transplantation of a healthy microbiome is not limited to clinical samples. An increasing interest in the transplantation of environmental (e.g., soil) microbiomes has been noted during the last couple of years for bioremediation purposes or to develop a specific environment of interest. These procedures are being developed, as this Special Issue is published, and there are many laboratories working on different aspects of how to use omics as a wonderful tool.

The application of multiomic approaches to identify therapeutic targets has been and is increasingly becoming considered. From genetic information to gene expression and function, a holistic perspective provides insights into therapeutic targets that would have been missed if using a single omics approach. While the human genome is home to thousands of genes and proteins, which represent a wide repertoire of potential therapeutic targets, the gut, and oral microbiomes are home to over 46 million bacterial genes, representing an even more extensive and widely unexplored repertoire for therapeutic targets that could also have the potential for personalized medicine. This has been particularly the case during the severe acute respiratory syndrome coronavirus 2 (SARS-CoV2) pandemic where genome, metagenome, and proteome information have not only helped to identify the potential nature and adaptation mechanisms of the virus, but are also aiding to identify associations between gut microbiota composition, levels of cytokines, and inflammatory markers in patients infected with SARS-CoV2. Results thus far suggest that the gut microbiome may be involved in the magnitude of disease severity.

However, we cannot become dogmatic and become so attached to the method that we forget the impact that treatments, such as those described above, may have on the individual or the environment. The Gaia Hypothesis clearly shows everything being connected, and we cannot obviate the ecology in any of these treatments/studies. In all cases, any ecosystem, including any we may have in plants, animals, and the environment, will have unique characteristics, and the microbiomes are likely to be in an equilibrium of some sort. Any outside forces will result in dysbiosis and a break in this equilibrium. We must not forget that although in equilibrium, all ecosystems are dynamic, and although plastic and flexible, intrinsic and extrinsic factors will have an impact on this equilibrium. Horizontal gene transmission is an intrinsic part of evolution; mobile elements, lysogenic phages, and even naked DNA will have an impact on the populations, leading in many cases to a break in the equilibrium; lysogenic phages and antibiotic-resistance genes come to mind. We have bacterial pathogens, such as O157:H7 an emerging pathogen that is the result of lysogenic conversion; in the presence of these lysogens in the population, the treatment of a patient could not include DNA-interfering antibiotics since this may result in the induction of lysogenic toxin gene-bearing coliphages that could actually increase the prevalence of these genes in *Escherichia coli* populations. Therefore, the role of bacteriophages as gene sequence vectors or any mobile elements in any given ecosystem needs to be thoroughly evaluated, and possible predictive models used to avoid antibiotic resistance or virulence factor transfer to a wider "bacterial audience". In addition, the fact that horizontal gene transmission occurs, and may, in fact, become more prevalent under traumatic conditions in the host (e.g., debridement in the case of skin wounds or use of antibiotics for gastrointestinal problems) needs to be taken into consideration.

Any complex problem, such as a microbial or viral-caused disease, demands a thorough understanding of all, or most, of the factors involved. We cannot think of a more complex problem than elucidating the dynamic interactions involving bacteria, archaea, viruses, protozoa, and host factors. The concept of VFARs (virulence factor activity relationships; a take on QSARS or quantitative structure activity relationships for chemicals) that has been used solely in the water quality research area as a possible means of predicting outbreaks and as a means of helping the Environmental Protection Agency in its search for possible Contaminant Candidate List additions, and as such is intriguing; however, the VFARs concept could also be used as a tool for an *a priori* analysis of the microbiota for microbial transplants. However, the presence of certain genes does not necessarily mean they are expressed, as shown by the presence of viral sequences not expressed in some cells. In any case, in order to ensure the safety of any microbial transplant, be it fecal, oral, or skin, we should take this concept into consideration in order to, *a priori*, know what possible risks there may be to the recipient. In any case, if we are to learn anything from previous mistakes, we should know that no single technique/method will give us all the answers. VFARs relying only on sequences will not allow us to see if any of the virulence factors are, in fact, capable of being expressed, of if they are just a part of the sequences. Therefore, a holistic multiomics approach can be extremely helpful, especially in the case of its possible use in microbial transplantation. 

The concept of One-Health also needs to be expanded and taken into consideration if public and environmental health are to be better understood and protected. The possibility that many virulence factors in human or animal pathogens may be derived from other ecosystems is open to debate and research. We are likely to have more emerging pathogens in the future as a result of the closer contact of humans to animals as well as encroachment into their habitats. The SARS CoV-2 may be an example of this, and may we not forget that many organisms have endogenous retroviruses as part of their genome as well as virusoids that may be present in the host as a result of past infections, and these may get the opportunity to be expressed resulting from co-infection with a helper virus. Multiomics give us an incredible manner of looking at gene sequences, gene expressions and potential horizontal gene transfer; let’s get on the bandwagon!

As the cost of applying various omic approaches decreases and the tools for data normalization, analysis, visualization, and cross-comparisons become increasingly available, we anticipate that the number of multiomic studies will continue to expand. Multiomic studies will continue to aid in the search for markers of health and disease and help explain biological phenomena from a holistic perspective. In this Special Issue, we have papers that have taken multiomics in all their potential.

